# The moderating role of mindfulness in the relationship between loneliness and depressive symptoms

**DOI:** 10.1038/s41598-024-81462-3

**Published:** 2024-12-28

**Authors:** Mei Chih Liao, Ming Sing Jessica Choi, Chak Hei Ocean Huang, Stanley Kam Ki Lam, Ming Yu Claudia Wong, Janet Yuen-Ha Wong, Hong Wang Fung

**Affiliations:** 1 Department of Psychology, California State University, Fullerton, USA; 2 Department of Psychology, The University of Hong Kong, Pokfulam, Hong Kong; 3 Department of Social Work and Social Administration, The University of Hong Kong, Pokfulam, Hong Kong; 4 Nethersole School of Nursing, Faculty of Medicine, The Chinese University of Hong Kong, Shatin, Hong Kong; 5 Department of Health and Physical Education, The Education University of Hong Kong, Tai Po, Hong Kong; 6 School of Nursing and Health Studies, Hong Kong Metropolitan University, Kowloon, Hong Kong

**Keywords:** Loneliness, Depression, Mindfulness, Moderator analysis, Public health, Human behaviour, Health care, Risk factors

## Abstract

Loneliness has been recognized as a pressing global health threat. Research shows that loneliness is associated with depressive symptoms, but less is known about what factors might influence this relationship. This study tested the hypothesis that mindfulness would buffer the association between loneliness and depressive symptoms. A total of 220 Chinese adults completed validated measures of loneliness, mindfulness, and depressive symptoms. Hierarchical multiple regression and moderator analyses were conducted. Mindfulness was significantly associated with depressive symptoms (β = -.485, *p*  < .001) after controlling for demographic variables and loneliness. Furthermore, mindfulness was a statistically significant moderator. Loneliness was more strongly associated with depressive symptoms when the levels of mindfulness were low. Mindfulness buffered the association between loneliness and depressive symptoms. Encouraging mindfulness practices might offer community-wide benefits from a public health perspective. While some studies showed that mindfulness could reduce loneliness, future studies should further evaluate whether mindfulness-based programs could also prevent the mental health consequences of loneliness.

## Loneliness as a pressing global health threat

Loneliness is an aversive psychological state when there is a discrepancy between one’s perception of their current and desired quantity and quality of interpersonal relationship^1^. Male gender, younger age, and individualistic cultures have been found to be associated with higher levels of loneliness^2^. In addition, because of potential sociocultural influences, research consistently shows significant differences in the prevalence of loneliness across cultures (e.g., 9.2% in South-East Asia, 14.4% in the Eastern Mediterranean region)^3^. Therefore, it is highly relevant to examine the correlates of loneliness across diverse sociocultural settings.

Given the robust association found between loneliness and poor physical and mental health, as well as mortality^4^, the World Health Organization^5^ has recently declared loneliness as a pressing global health threat. Researchers have observed the close association between loneliness and mental health and behavioral problems, including depression^6^ and smoking^7^.

## The relationship between loneliness and depressive symptoms

One of the most established relationship between prolonged loneliness and mental health is an increased risk of depression for a meta-analysis, see^6^. Depression is an affective disorder characterized by depressed mood, loss of interest, feeling of worthlessness, psychomotor agitation and sleep disturbances^8^, which significantly decreases quality-adjusted life expectancy by 28.9 years^9^. Even subclinical level of depression has been demonstrated to substantially increase the economic costs by USD160-192 million per 1 million inhabitants^10^, highlighting its significant burden on the individual and the society.

Recently, a bidirectional connection between loneliness and depression has been demonstrated. According to the affiliation motive model (RAM), people in transient loneliness tend to negatively interpret social information and withdraw from social situations as an act of behavioral confirmation after feeling lonely^11^, and this self-reinforcing loop could increase the extent and duration of loneliness^12^. It suggests that loneliness promotes the development of maladaptive behaviors, which could in turn increase the feeling of loneliness and psychological distress^13^. Therefore, loneliness could possibly lead to depressive symptoms^14,15^.

## The present study: exploring the moderating role of mindfulness

In order to inform preventive strategies, a few studies have tried to identify factors that could influence or moderate the relationship between loneliness and depression, such as social support^16^, self-esteem^17^, and socio-economic status^18^. Different approaches have been implemented in an attempt to alleviate loneliness, and one of the most promising interventions is mindfulness-based intervention which has been consistently demonstrated to improve loneliness^19,20^. During the practice of mindfulness, one learns to purposefully pay attention in a particular way, constructing the core axioms of mindfulness by ‘intention’, ‘attention’ and ‘attitude’, leading to the stage of reperceiving and thus a shift in perspective^21^. This meta-mechanism of action builds the basis of self-regulation, values clarification, increased cognitive, emotional and behavioral flexibility as well as exposure to overwhelming emotions, thoughts or sensations^21^. This allows individuals to gain an awareness that they have control over their own thoughts and emotions, and that their self-identities are separated from distress, decreasing the duration of negative affect and thus risks of depression^22^. Besides, paying attention to the present moment nonjudgmentally could increase openness and acceptance towards one’s experience, fostering the equanimity with loneliness and social isolation, thus encouraging social engagement^20^, which could prevent depression and promote recovery^23^.

Besides psychological mechanisms, evidence has shown support to the neurobiological basis underlying the benefits of mindfulness: Mindfulness processing involves greater activation in the anterior cingulate cortex, insular cortex and prefrontal cortex, as well as a deactivation of the amygdala, which are responsible for emotional regulations and a decrease in self-referential processing^24^. By encouraging a focus on present sensory experiences, one could detach from self-referential processing that could lead to rumination^24^. Therefore, informed by this literature, it is believed that mindfulness has the potential to buffer the relationship between loneliness and depressive symptoms.

Although mindfulness-based interventions have been increasingly recognized as a potentially effective way to reduce loneliness^25^, little is known about whether mindfulness would be a significant moderator. One recent Australian study (N = 297) found that loneliness was significantly associated with psychological distress only in participants with low levels of mindfulness^26^. While this study is important, we were not aware of other studies that examined the moderating role of mindfulness in the relationship between loneliness and mental health outcomes. Considering the fact that there is a replication crisis in the psychology literature, findings from one single study may not be convincing enough. Therefore, more studies are needed to test the moderating effect of mindfulness. In addition, potential cultural differences should be considered when investigating mental health issues across the world, given that cultures may influence the perception or experience of loneliness^2^, the understanding of mental health problems^27^, as well as how people cope with distress and mental health struggles^28^. Therefore, we aimed to replicate the findings of the Australian study using a Chinese sample to improve our understanding of the role of mindfulness in the relationship between loneliness and mental health outcomes by testing the hypothesis that mindfulness would moderate the relationship between loneliness and depressive symptoms.

## Methods

### Participants

The study analyzed data from a mental health survey project, which obtained ethical approval at the Hong Kong Baptist University. The study was conducted in accordance with the relevant guidelines and regulations, including the Declaration of Helsinki. Potential participants were recruited using online advertising on Facebook and Instagram in 2023. To meet the inclusion criteria, participants had to be aged 18 or above, able to read and write Chinese, and agree to provide informed consent. Participants were excluded if they self-reported having a diagnosed reading disorder, dementia or intellectual disabilities.

## Measures

The online survey included a set of questions regarding demographic characteristics, such as age, gender and education level, in addition to validated measures of loneliness, mindfulness, and depressive symptoms.

*UCLA 3-Item Loneliness Scale.* The UCLA 3-Item Loneliness Scale is a brief adaptation of the UCLA Loneliness Scale^29,30^. This self-report measure assesses loneliness through three items. Higher scores indicate higher levels of loneliness. The Chinese version of the UCLA 3-Item Loneliness Scale^31,32^ was used in the present study (Cronbach’s alpha = 0.862).

*Patient Health Questionnaire-9 (PHQ-9).* The PHQ-9 is a self-administered screening tool consisting of 9 items that evaluate depressive symptoms experienced over the past two weeks^33^. Responses range from 0 (“Not At All”) to 3 (“Nearly Every Day”), with higher scores representing more severe depressive symptoms. The Chinese version of the PHQ-9^34^ was used in the present study (Cronbach’s alpha = 0.916).

*Mindful Attention Awareness Scale (MAAS).* The MAAS is one of the most widely used measures of trait mindfulness^35^ and is a reliable and valid self-report measure of mindfulness^36^. The MAAS is originally a 15-item, 6-point Likert scale designed to assess mindfulness through statements regarding everyday experiences. On this scale, responses vary from 1 (“Rarely”) to 6 (“Almost Always”), with higher total scores indicating higher levels of mindfulness after reverse scoring. The Chinese version of the MAAS was found to be reliable and valid^37^. The five items with the highest factor loadings (items 3, 7, 8, 10, and 14) were selected for use in the present study (Cronbach’s alpha = 0.907).

## Data analysis

Data analysis was performed using SPSS 22.0. We first reported the descriptive statistics of the sample. Subsequently, Pearson and point-biserial correlation analyses were conducted to identify the correlates of depressive symptoms. Finally, hierarchical multiple regression and moderator analysis using the SPSS PROCESS V3.2 macro^38^ were conducted to examine whether mindfulness levels would moderate the relationship between loneliness and depressive symptoms.

## Results

### Sample characteristics

The study included 220 participants with an average age of 32.3 years (SD = 14.3). The sample was predominantly female (80%), with 69% holding at least a university degree. Most participants lived in Taiwan (69.1%), Hong Kong (27.3%), or Macao (2.3%). The mean PHQ-9 score was 11.63 (SD = 7.52); 59.5% screened positive for depression (PHQ-9 ≥ 10). The sample characteristics are reported in Table 1.

**Table 1:** Sample characteristics and correlates of depressive symptoms (N = 220).

Variables	Mean (SD) / Frequency (%)	Correlation with depressive symptoms
Age	32.3 (14.3)	-.34***
Gender (Female)	176 (80.0%)	.08
Education (Undergraduate or above)	151 (68.6%)	.01
Depressive symptoms (possilbe range: 0 to 27)	11.6 (7.52)	/
Loneliness (possible range: 3 to 12)	7.28 (2.79)	.54***
Mindfulness (possilbe range: 5 to 30)	19.8 (6.66)	-.63***

*p < .05 **p < .01 ***p < .001.

## The relationship between loneliness, mindfulness, and depressive symptoms

As reported in Table 1, depressive symptoms were significantly positively related to loneliness (r = 0.54, p < 0.001), while negatively correlated with mindfulness (r = -0.63, p < 0.001) and age (r = -0.34, p < 0.001).

After controlling for demographic variables and taking loneliness into account, mindfulness was significantly associated with depressive symptoms (β = -0.485, p < 0.001) (see Table 2).

**Table 2:** Hierarchical multiple regression predicting depressive symptoms (N = 220).

Variables	Depressive symptoms							
	B	SE	β	p	F	Adjusted R^2^	ΔR^2^	ΔF
Step 1					9.587***	.105	.118	9.587***
Age	-.180	.035	-.343	< .001				
Gender (Female)	.389	1.219	.021	.750				
Education (Undergraduate or above)	-.699	1.048	-.043	.505				
Step 2					42.429***	.486	.380	81.035***
Age	-.027	.029	-.052	.343				
Gender (Female)	-1.482	.936	-.079	.115				
Education (Undergraduate or above)	-.305	.795	-.019	.701				
Loneliness	.908	.148	.336	< .001				
Mindfulness	-.549	.063	-.485	< .001				
Step 3					37.019***	..497	.013	5.504*
Age	-.034	.029	-.065	.232				
Gender (Female)	-1.463	.926	-.078	.116				
Education (Undergraduate or above)	-.173	.789	-.011	.826				
Loneliness	1.737	.383	.643	< .001				
Mindfulness	-.249	.142	-.220	.082				
Interaction term	-.044	.019	-.332	.020				

*p < .05 **p < .01 ***p < .001.

## The moderating role of mindfulness

A multiple regression model was further conducted to investigate whether the association between loneliness and depression would depend on levels of mindfulness. We found that the interaction term (loneliness*mindfulness) was a significant predictor of depressive symptoms (β = -0.332, p = 0.020), ΔR^2^ = 0.013, ΔF = 5.504, p < 0.05 (see Table 2).

Subsequently, we conducted moderation analysis using SPSS PROCESS Model 1 to assess the moderating role of mindfulness. As summarized in Table 3, loneliness was more strongly associated with depressive symptoms when the levels of mindfulness were lower.

**Table 3:** Conditional effects of the focal predictor (loneliness) on depressive symptoms at values of the moderator (N = 220).

Mindfulness	Effect	SE	t	p	LLCI	ULCI
Low (13.1145)	1.1539	.1815	6.3578	< .001	.7961	1.5116
Mid (19.7727)	.8582	.1470	5.8385	< .001	.5685	1.1479
High (26.4309)	.5625	.2029	2.7730	< .001	.1627	.9624

The cutoffs of the moderator were generated from SPSS PROCESS Model 1 for probing the details of interaction and they do not have implications on groupings.

**Fig. 1:**
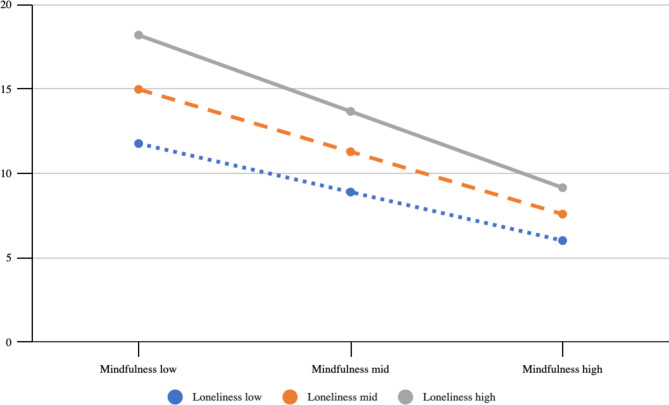
Conditional effects of loneliness on depressive symptoms, with mindfulness as a moderator and age as a covariate Notes. The plot was generated in SPSS PROCESS Model 1 to visualize the conditional effect of the focal predictor. The cutoffs of the independent variable and the moderator were generated from SPSS PROCESS Model 1 for probing the details of interaction and they do not have implications on groupings. The Y axis was calibrated as the possible scores of the dependent variable (PHQ-9), which may range from 0 to 27.

Simple slopes for the association between loneliness and depression were tested for low (-1 SD below the mean), moderate (mean), and high (+ 1 SD above the mean) levels of mindfulness. Each of these tests revealed a significant outcome, which indicates the positive relationship between loneliness and depression. Loneliness was more strongly related to depression for lower levels of mindfulness (B = 1.15, t (215) = 6.34, p < 0.001) or moderate (B = 0.85, t (215) = 5.75, p < 0.001) than high (B = 0.56, t (215) = 2.20, p = 0.03) levels of mindfulness (see Table 3 and Fig. 1).

To be more precise, using the Johnson-Neyman technique^39^, which assesses how a continuous variable moderates the relationship between an independent variable and the dependent variable, we found that when the levels of mindfulness were very high (MAAS > 28.7), the relationship between loneliness and depression were no longer significant.

## Discussion

This study replicated the observation of a positive association between loneliness and depressive symptoms, indicating that as loneliness increases, depressive symptoms rise in our sample of Chinese adults. Additionally, consistent with the results of one recent Australian study^26^, we found that mindfulness serves as a significant moderator in the relationship between loneliness and mental health outcomes (in particular, depressive symptoms). The strength of the relationship between loneliness and depression decreases as the levels of mindfulness increase.

In the present study, the relationship between loneliness and depression was successfully replicated in our non-western sample, which is aligned with existing evidence^6^. We provide novel findings showing that mindfulness acts as a moderator in this relationship. The results contribute to the ongoing body of knowledge regarding how to better prevent or buffer the mental health issues related to loneliness. Given the moderating role of mindfulness, the potential benefits of mindfulness-based interventions in alleviating the negative impacts of loneliness should receive more attention. Future studies should further investigate the mechanisms and the causal relationship behind the effects of mindfulness on perceived loneliness and depressive symptoms, such as its role in neuroplasticity^40^ and cognitive functioning^41^, as well as whether it has an advantage over other interventions in terms of effectiveness and cost–benefit.

As loneliness has been an increasingly significant public health concern^5^, policymakers and health and social service providers should implement evidence-informed strategies to alleviate its negative mental health effects. Given the buffering effects of mindfulness, as indicated in the recent study published by Coutts-Smith and Phillips^26^ as well as in our current study, large-scale community-based mindfulness training program might be a potentially effective approach. As loneliness is a significant predictor of subsequent depressive symptoms, including among older adults^42^, it may be helpful to encourage people experiencing loneliness or social isolation to practice mindfulness in daily life. Further studies are also needed to evaluate the potential effectiveness of large-scale mindfulness training programs^43^.

This study has the strengths of utilizing well-validated measures and employing a non-Western sample. Yet, there are also several limitations. First, our sample was not representative of the general population. However, it should be noted that health-related studies that aim to test hypotheses or look for relationships among variables are usually conducted using convenience samples, and representative samples are not necessary^44^. The findings of our study are meaningful and can contribute to the limited literature on protective factors in loneliness and mental health problems . Second, the cross-sectional design of this study did not allow us to examine the direction of the relationship. Future studies can further examine the moderators in the relationship between loneliness and mental health outcomes using longitudinal data. Third, while the Mindful Attention Awareness Scale (MAAS) is one of the most-commonly used measures of trait mindfulness, the items are worded negatively or describe the absence of attentional focus^45^. Another limitation regarding the use of MAAS is that it is a single factor measure of trait mindfulness focusing on self-awareness. The MASS may not be able to capture different dimensions of mindfulness. Therefore, future studies should replicate our findings using alternative and more comprehensive measures of mindfulness. Fourth, while this observational study provides some insightful findings, future research should use experimental designs (e.g., controlled trials) to further examine the benefits of mindfulness.

## Data Availability

Data that support the findings of this study are available from the corresponding authors upon reasonable request.
